# Knowledge, Attitudes, and Behaviors Regarding Laser Hair Removal of Medical Students: A Cross‐Sectional Study

**DOI:** 10.1111/jocd.71173

**Published:** 2026-09-30

**Authors:** Emrah Atay, Hilal Kaya Erdoğan, Sibel Sert, Büşra Yeni Yıldız, Esra Ağaoğlu, Selma Metintaş

**Affiliations:** ^1^ Department of Public Health, Faculty of Medicine Eskişehir Osmangazi University Eskişehir Turkey; ^2^ Department of Dermatology and Venereology, Faculty of Medicine Eskişehir Osmangazi University Eskişehir Turkey; ^3^ Eskişehir Yunus Emre State Hospital Eskişehir Turkey

**Keywords:** attitudes, behaviors, knowledge, laser hair removal, medical education, medical students

## Abstract

**Introduction:**

Laser hair removal (LHR) has become one of the most common procedures among non‐invasive cosmetic treatments. The structured training on cosmetic dermatological procedures may be limited during medical education. The aim of this study is to evaluate medical students' knowledge, attitudes, and practices regarding LHR.

**Materials and Methods:**

This cross‐sectional study was conducted with 973 medical students at a medical school. They were asked to complete a questionnaire related to the history of LHR, sources of information about LHR and 12 statements in a 3‐point Likert scale format to assess students' knowledge levels named “LHR‐K‐Scale”. As the data were not normally distributed, non‐parametric tests (Mann–Whitney U and Kruskal–Wallis) were used for group comparisons. Univariate and multivariable binary logistic regression analyses were performed to identify factors associated with sufficient knowledge.

**Results:**

Of the students, 484 (49.7%) were female and 489 (50.3%) were male; their ages ranged from 17 to 31, with a mean ± SD of 20.9 ± 2.2 years. The total LHR‐K Scale score ranged from 0 to 24, with a mean score of 13.5 ± 6.5. Overall, 527 students (54.2%) were classified as having a sufficient level of knowledge (≥ 15 points). In the logistic regression analysis, correctly knowing the definition of LHR increased the likelihood of having a sufficient level of knowledge on the LHR‐K Scale by 2.50 times (OR = 2.50; 95% CI: 1.90–3.30). Participants who had undergone LHR were 1.98 times more likely to have a sufficient level of knowledge than those with no history of LHR and no intention to undergo the procedure (OR = 1.98; 95% CI: 1.37–2.87). In addition, participants who used social media as a source of information were 1.54 times more likely to have a sufficient level of knowledge than those who did not (OR = 1.54; 95% CI: 1.15–2.05).

**Conclusions:**

The study revealed that while medical students' knowledge levels regarding LHR are heterogeneous, they are generally limited; significant knowledge gaps exist in areas such as mechanism of action, radiation, pregnancy, breastfeeding, cancer, and infertility. The topic should be addressed not only as a matter of individual aesthetic preference but also from the perspectives of health literacy. It is recommended that the medical curriculum include up‐to‐date and evidence‐based content regarding cosmetic dermatology practices, particularly LHR.

## 1 Introduction

In recent years, global demand for cosmetic procedures has been rapidly increasing, reflecting not only technological advancements but also the growing commercialization of healthcare services and shifting societal perceptions of beauty. Laser hair removal (LHR) has become one of the most common procedures among non‐invasive cosmetic interventions. In line with this trend, an international study has reported that *“hair removal”* procedures rank among the top five non‐surgical aesthetic procedures. According to the same report, *“hair removal”* procedures also rank among the top non‐surgical applications in Türkiye [1]. Similarly, findings from the American Society of Dermatologic Surgery Consumer Survey indicated that laser hair removal (LHR) was among the top five most preferred cosmetic procedures in 2025 [2].

LHR targets hair follicles using the principle of selective photothermolysis and is considered safe when performed under appropriate conditions. However, adverse events reported in the literature such as burns, pigmentation disorders, paradoxical hair growth, and ocular damage are often associated with non‐medical practitioners who lack proper authorization and expertise, inadequate training, inappropriate patient selection [3, 4].

Advances in laser technology, an improved safety profile across different skin phototypes, and shorter procedure times have significantly expanded the method's clinical applicability [5]. Furthermore, the widespread adoption of the process and the increasing influence of social media‐driven beauty standards appear to be associated with the growing demand for these procedures [6, 7].

Similar to other cosmetic procedures, the increasingly blurred boundaries of LHR within the healthcare system and its provision outside traditional clinical settings in a broader commercial context have raised new public health debates regarding safe practice standards, ethical responsibilities, and the societal impacts of preventable complications [8]. Driven by digital media and commercial advertising, the dissemination of information regarding cosmetic procedures has accelerated; however, it has also facilitated the spread of unsubstantiated or incomplete information [9, 10, 11]. Insufficient health literacy may limit individuals' ability to adequately assess potential risks and is associated with engagement in unsafe practices [12]. Therefore, raising awareness in the community is a crucial strategy for reducing preventable harms and supporting informed decision‐making processes.

It has been reported that structured training in cosmetic dermatological procedures is often limited, particularly at the undergraduate level; this situation may create a gap between rapid technological advancements in the cosmetic field and physicians' competencies in consultation and risk assessment [13, 14]. Assessing medical students' knowledge and awareness levels regarding LHR is important not only for identifying individual knowledge gaps but also for informing improvements in health education and supporting the development of community‐based preventive strategies. Therefore, this study aimed to assess medical students' knowledge, attitudes, and practices regarding LHR, as well as their utilization of these procedures at a university medical school.

## 2 Material and Method

### 2.1 Study Design and Sample

This is a cross‐sectional study conducted among medical students during the 2023–2024 academic year. The medical school where the study was conducted (Eskişehir Osmangazi University Faculty of Medicine) had a total of 1792 students during the 2023–2024 academic year. The required sample size was calculated using the Epi Info program, assuming a population of 1792, a LHR procedure rate of 50%, a margin of error of 4%, a 95% confidence interval, and a design factor of 2.0, resulting in a minimum of 900 participants. Students were randomly assigned to the study group, taking into account the weight of the number of students in each semester. A total of 973 students comprised the study group.

### 2.2 Data Collection

For this study, a questionnaire was developed based on the literature [4, 13, 15]. The questionnaire consisted of four sections. The first section covered sociodemographic characteristics (grade level, age, gender, place of residence, family income), the second section included questions regarding sources of information about LHR (awareness of LHR, sources of information, use of social media to access information about LHR), and the third section included questions related to the history of undergoing LHR (history of undergoing LHR, consideration of undergoing LHR, reasons for not undergoing LHR, levels of concern regarding LHR). The fourth section of the survey included knowledge questions designed to assess students' knowledge of LHR, consisting of 12 statements—five of which were false—presented on a 3‐point Likert scale (agree/disagree/undecided).

After informing participants about the study's purpose, consent was obtained. The survey forms were completed independently by the participants under observation. The surveys were distributed to prevent data loss and collected immediately afterward. This process took approximately 15 min.

### 2.3 Development of the Laser Hair Removal Knowledge Level Assessment Tool

A pool of items for the LHR knowledge questions (LHR‐K‐Scale) was created by two experienced dermatologists based on the literature. The items were then evaluated by 10 experts for content validity. Experts were asked to assess each item as *“essential,” “unnecessary,”* or “essential but needs revision.” The Content Validity Index (CVI) was calculated as 0.80 by taking the average of the Content Validity Ratio (CVR) values of the items in the item pool. Since the CVI value was greater than 0.60, it was considered statistically significant, and the scale's content validity was deemed adequate [16]. Subsequently, one language expert and two experts in measurement and evaluation reviewed the items for clarity and readability, and necessary revisions were made. A pilot study was conducted with 21 participants prior to the main study to assess the reliability of the scale. In the pilot study, Cronbach's alpha coefficient was 0.777. All item–total correlation coefficients were above the acceptable threshold (0.25) [17].

### 2.4 Statistical Analysis

The analysis of the obtained data was conducted using the SPSS (version 24.0) and JASP statistical software packages. The validity of the LHR‐K‐Scale was assessed through exploratory factor analysis (EFA) and item‐total correlation coefficients. Reliability was evaluated using Cronbach's alpha, which was 0.853, indicating high internal consistency. Item‐total correlations ranged from 0.388 to 0.612. Multicollinearity was ruled out as inter‐item correlation coefficients did not exceed 0.800.

To determine the suitability of the data for factor analysis, the Kaiser‐Meyer‐Olkin (KMO) measure of sampling adequacy and Bartlett's test of sphericity were employed. The KMO value was 0.846, and Bartlett's test was statistically significant (*X* ^2^ = 5080.914; *p* < 0.001), confirming the sample size was sufficient for EFA. In the Exploratory Factor Analysis of the LHR‐K‐Scale, item analysis was conducted using principal component analysis. A factor loading cutoff value of 0.40 (min‐max: 0.415–0.686) was accepted. The resulting single‐factor model explained 33.2% of the cumulative variance. To assess the scale's item discrimination, the presence of a significant difference in item scores between the lower and upper 27% groups indicated that the measurement tool possessed sufficient discriminatory power (*p* < 0.001). Following analyses conducted to evaluate the validity of the LHR‐K‐Scale, the measurement tool was deemed sufficiently valid, and the data analysis proceeded.

The items assessing knowledge were answered using the response options “true,” “false,” and “I don't know.” Scoring was assigned as 2 points for a correct response, 1 point for “I don't know,” and 0 points for an incorrect response. This scoring system was not intended to treat the “I don't know” response as equivalent to correct knowledge; rather, it was designed to distinguish between correct knowledge, awareness of one's lack of knowledge, and misinformation. Thus, recognizing one's lack of knowledge was conceptually differentiated from holding incorrect knowledge. The total score that can be obtained from the LHR‐K‐Scale ranges from 0 to 24. Each participant's total score was calculated, converted into percentages to classify their knowledge levels, and evaluated using Bloom's cutoff criteria. While previous literature has utilized three‐tier systems (good, moderate, poor), this study adopted a 60% cutoff point to ensure a balanced distribution and enhance statistical power. Accordingly, participants who scored 60% or higher (≥ 15 points) were grouped as having an “adequate/good knowledge level”, while those scoring below 60% were grouped as having an “inadequate/poor knowledge level” [18, 19, 20].

Participants were asked to self‐rate their level of concern regarding LHR using a 10‐point Visual Analog Scale (VAS), ranging from 0 (“no concern at all”) to 10 (“extreme concern”). The VAS is a single‐item instrument commonly used to assess perceived anxiety or concern related to medical procedures [21]. Participants independently selected the score that best reflected their own level of concern regarding LHR; no assessment was made by the researchers. The distribution of VAS scores in the entire study population was examined, and the 75th percentile was identified as 4. Accordingly, participants with a VAS score of ≥ 4 were classified as having *“high concern”*.

Grade level was categorized as pre‐clinical (years 1–3) and clinical (years 4–6) based on the structure of the medical curriculum. Family income level was assessed using participants' self‐reported perception of their family's economic status. Participants categorized their family income as low, medium, or high according to their own assessment.

As the data were not normally distributed, non‐parametric tests (Mann–Whitney U and Kruskal–Wallis) were used for group comparisons, as appropriate. Univariate and multivariable logistic regression analyses were performed to identify factors associated with sufficient knowledge. Variables with a *p*‐value < 0.05 in univariate analyses were included in the multivariate model. Results were reported as crude odds ratios (CORs) and adjusted odds ratios (AORs) with 95% confidence intervals (CIs). A *p*‐value of ≤ 0.05 was considered statistically significant.

## 3 Results

Of the 973 medical students comprising the study group, 484 (49.7%) were female and 489 (50.3%) were male. Their ages ranged from 17 to 31, with a mean ± SD of 20.9 ± 2.2 years. The distribution of the study group's sociodemographic characteristics is presented in Table 1.

**TABLE 1:** Sociodemographic characteristics of the participants.

Sociodemographic characteristics	*n* (%)
Age, mean ± SD	20.9 ± 2.2
Gender	
Male	489 (50.3)
Female	484 (49.7)
Grade (year of study)	
Pre‐clinical (years 1–3)	565 (58.1)
Clinical (years 4–6)	408 (41.9)
Place of residence	
City center	714 (73.4)
Town/village	259 (26.6)
Family income level	
Low	67 (6.9)
Medium	812 (83.5)
High	94 (9.6)
Total	973 (100.0)

Overall, 556 (57.1%) participants correctly answered the question “What is laser hair removal?” The questions with the highest percentage of correct answers regarding LHR among the study group were, in order: “After laser hair removal, the treated area should be protected from the sun,” “Protective eyewear should be worn during laser hair removal,” and “Laser hair removal should not be performed on children under 12 years of age.” The least correctly answered statement was “Laser hair removal is a definitive solution for the permanent removal of hair.” The distribution of responses to statements regarding laser hair removal among the study group is presented in Table 2.

**TABLE 2:** Distribution of responses to statements on the LHR‐K Scale.

Statements	Correct *n* (%)	Incorrect *n* (%)	Undecided/Don't know *n* (%)
1. The mechanism of action of laser hair removal involves the absorption of laser light by melanin in the hair follicle and the destruction of the hair follicle. (T)	652 (67.0)	21 (2.2)	300 (30.8)
2. Laser hair removal is a definitive solution for permanent hair removal. (T)	147 (15.1)	606 (62.3)	220 (22.6)
3. Laser hair removal emits radiation. (F)	466 (47.9)	147 (15.1)	360 (37.0)
4. Laser hair removal should not be performed during pregnancy. (T)	440 (45.2)	147 (15.1)	386 (39.7)
5. Laser hair removal should not be performed during breastfeeding. (T)	386 (39.7)	145 (14.9)	442 (45.4)
6. Laser hair removal should not be performed on children under 12 years of age. (T)	657 (67.5)	55 (5.7)	261 (26.8)
7. Laser hair removal may cause skin cancer. (F)	239 (24.6)	268 (27.5)	466 (47.9)
8. Laser hair removal applied to the genital area may cause infertility. (F)	483 (49.6)	112 (11.5)	378 (38.9)
9. Laser hair removal on the underarms may damage lymph nodes and cause lymphoma. (F)	340 (34.9)	208 (21.4)	425 (43.7)
10. Laser hair removal may cause breast cancer. (F)	388 (39.9)	143 (14.7)	442 (45.4)
11. Protective eyewear should be worn during laser hair removal. (T)	708 (72.8)	47 (4.8)	218 (22.4)
12. After laser hair removal, the treated area should be protected from the sun. (T)	723 (74.3)	27 (2.8)	223 (22.9)

Abbreviations: F, false statement; T, true statement.

The total score on the LHR‐K‐Scale ranged from 0 to 24, with a mean score of 13.5 ± 6.5 and a median of 15 (Q1–Q3: 10–18.5). According to Bloom's cutoff criteria, 527 students (54.2%) had an adequate level of knowledge (≥ 15 points).

Among the participants, 314 students (32.3%) had undergone LHR. Among those who had not, 46.1% (304) stated that they did not intend to undergo the procedure in the future. The primary reason for not undergoing LHR was a lack of sufficient information about the procedure, cited by 48.4% of respondents. Barriers to undergoing laser hair removal among the study participants are presented in Figure 1.

**FIGURE 1:**
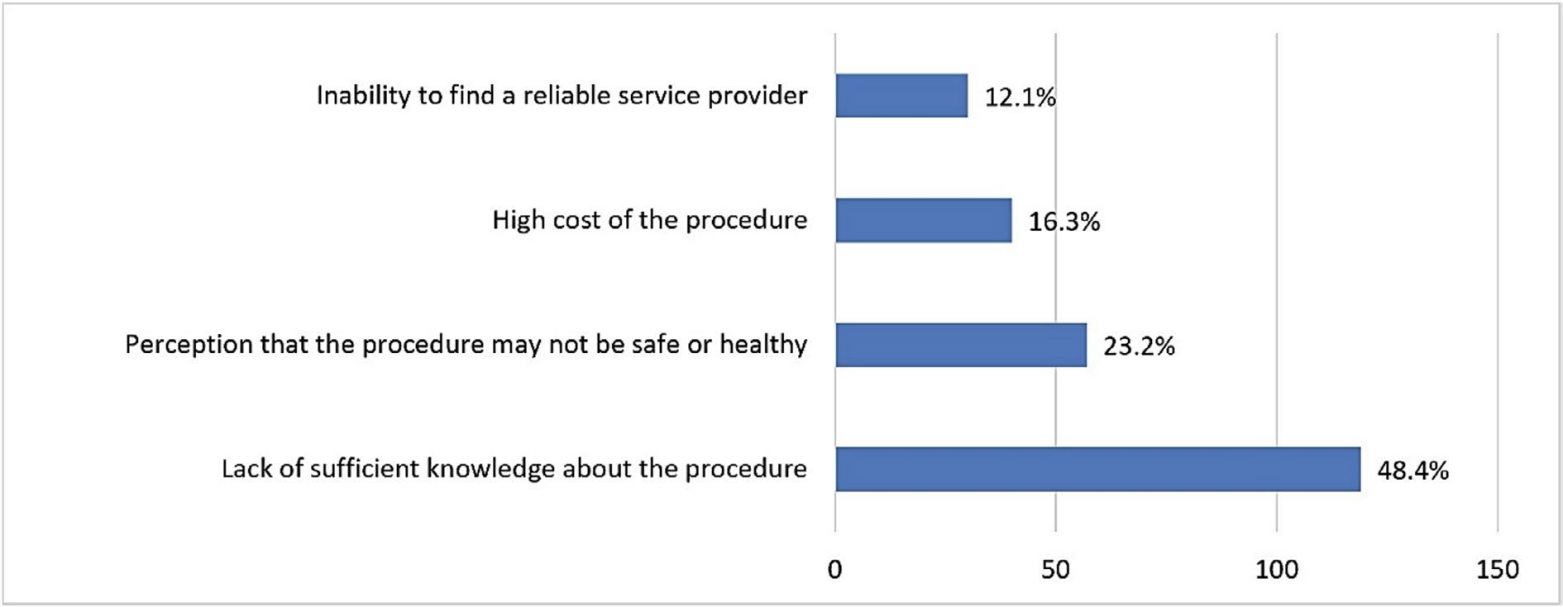
Barriers to undergoing laser hair removal. Percentages were calculated based on the total number of responses provided by participants.

Regarding sources of information, 54.4% of students reported using the internet, 53.5% social media, and 53.1% relatives or friends, whereas only 25.5% reported consulting healthcare professionals. The distribution of LHR‐K scores according to information sources is shown in Figure 2.

**FIGURE 2:**
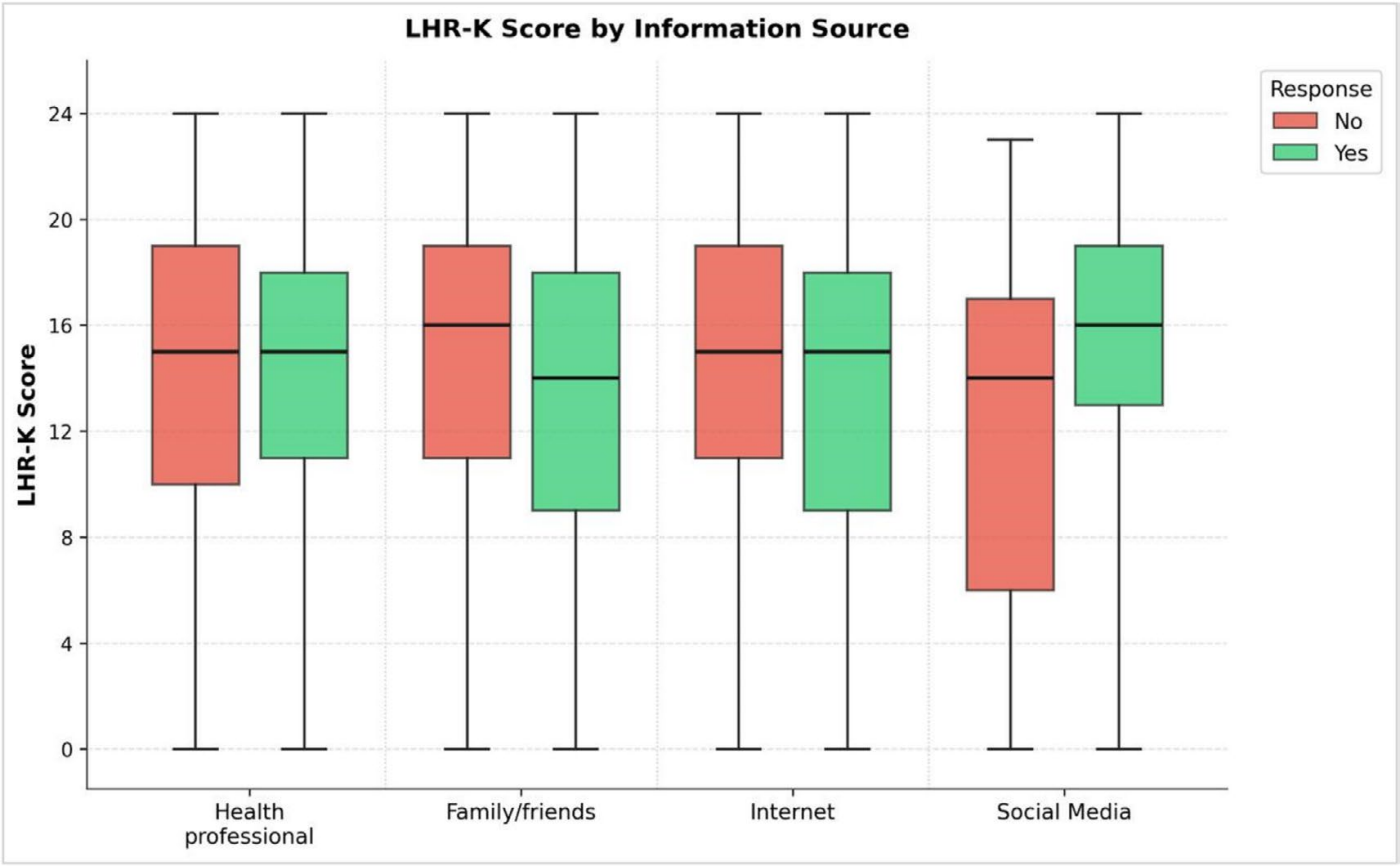
Distribution of LHR‐K scores according to information sources.

Overall, 385 participants (39.6%) reported having concerns regarding LHR. Among those reporting concern, the median VAS score was 5 (Q1–Q3: 4–7). Based on the predefined cut‐off (VAS ≥ 4), 293 participants (30.1% of the total sample) were classified as having a high level of concern. Participants who correctly answered the statements regarding radiation exposure, pregnancy, breastfeeding, skin cancer, infertility, lymphoma, and breast cancer (Items 3, 4, 5, and 7–10) reported significantly lower levels of concern regarding LHR than those who answered these statements incorrectly or reported being undecided (all *p* < 0.05).

Participants with a history of LHR had significantly higher knowledge scores (median: 17, Q1–Q3: 14–20) than those without previous experience (median: 13, Q1–Q3: 4–17, *p* < 0.001). Among participants without a history of LHR, those considering undergoing the procedure had higher knowledge scores (median: 15, Q1–Q3: 9–18) than those who did not intend to undergo LHR (median: 13, Q1–Q3: 4–17, *p* < 0.001). Furthermore, participants who reported insufficient information as a reason for not intending to undergo LHR had substantially lower LHR‐K scores (median: 9, Q1–Q3: 0–16) than those who reported other reasons (median: 14, Q1–Q3: 8–18; *p* < 0.001). No statistically significant differences in LHR‐K scores were observed according to grade, gender, place of residence, family income level, or obtaining information from healthcare professionals (*p* > 0.05).

Participants obtaining information through social media had higher knowledge scores (median: 16, Q1–Q3: 13–19) than those who did not use social media as an information source (median: 14, Q1–Q3: 6–17, *p* < 0.001). Participants who obtained information from the internet differed significantly from those who did not; however, the median scores were similar between the groups (median: 15, Q1–Q3: 11–19 vs. median: 15, Q1–Q3: 9–18; *p* = 0.029). Participants who did not obtain information from relatives or friends also had higher knowledge scores (median: 16, Q1–Q3: 11–19) than those who did (median: 14, Q1–Q3: 9–18, *p* < 0.001). Participants with low concern regarding LHR had higher knowledge scores than those with high concern (median: 15, Q1–Q3: 10–19 vs. median: 14, Q1–Q3: 10–17, *p* = 0.011).

In univariate analyses, participants who had undergone LHR, those who had not undergone LHR but intended to undergo the procedure, those reporting lower levels of concern regarding LHR, and those obtaining information from social media, the internet, or relatives and friends were more likely to have sufficient knowledge. No statistically significant associations were found between sufficient knowledge regarding LHR and age, gender, grade level, place of residence, family income level, or obtaining information from healthcare professionals.

In the multivariable logistic regression analysis, correct knowledge of the definition of LHR (AOR = 2.50; 95% CI: 1.90–3.30), having undergone LHR (AOR = 1.98; 95% CI: 1.37–2.87), and obtaining information through social media (AOR = 1.54; 95% CI: 1.15–2.05) remained independently associated with sufficient knowledge, whereas obtaining information from the internet, obtaining information from relatives or friends, and level of concern regarding LHR did not (Table 3).

**TABLE 3:** Univariate and multivariate logistic regression analysis of factors associated with sufficient knowledge of LHR among medical students.

Variables	*n* (%)	Univariate COR (95% CI)	Multivariate AOR (95% CI)
Age, years	—	0.99 (0.93–1.05)	—
Gender (ref: female)			
Male	489 (50.3)	1.05 (0.82–1.36)	—
Grade (ref: clinical)			
Pre‐clinical	565 (58.1)	1.19 (0.92–1.53)	—
Place of residence (ref: town/village)			
City center	714 (73.4)	1.20 (0.90–1.60)	—
Family income level (ref: low)			
Medium	812 (83.5)	1.07 (0.65–1.76)	—
High	94 (9.6)	0.67 (0.36–1.25)	—
LHR experience/intention status (ref: no history and no intention)			
No history but intends to undergo LHR	355 (36.5)	1.66 (1.22–2.26)*	1.35 (0.96–1.89)
Had undergone LHR	314 (32.3)	3.24 (2.33–4.52)**	1.98 (1.37–2.87)**
Correct knowledge of LHR definition (ref: no)			
Yes	556 (57.1)	3.12 (2.40–4.06)**	2.50 (1.90–3.30)**
Information source: social media (ref: no)			
Yes	521 (53.5)	1.98 (1.54–2.56)**	1.54 (1.15–2.05)*
Information source: internet (ref: no)			
Yes	529 (54.4)	1.63 (1.15–2.30)*	1.14 (0.83–1.57)
Information source: healthcare professional (ref: no)			
Yes	248 (25.5)	1.03 (0.83–1.13)	—
Information source: relatives/friends (ref: no)			
Yes	517 (53.1)	1.78 (1.26–2.50)*	1.33 (0.97–1.83)
Level of concern regarding LHR (ref: high concern)			
Low concern	682 (70.1)	1.33 (1.01–1.76)*	1.27 (0.94–1.71)

Abbreviations: AOR, adjusted odds ratio; CI, confidence interval; COR, crude odds ratio; LHR, laser hair removal; Ref, reference category.

* Statistically significant at *p*‐value < 0.05.

** Statistically significant at *p*‐value < 0.001.

## 4 Discussion

LHR is one of the most commonly used cosmetic dermatology procedures today, and the current literature indicates that this method is generally safe when performed under appropriate indications, with the correct device and parameter selection, and by a trained practitioner. Adverse effects reported in the literature are mostly local and transient reactions such as erythema, perifollicular edema, and pigment changes, with a low rate of permanent sequelae; furthermore, no convincing evidence of risk, particularly regarding carcinogenicity, has been demonstrated regarding the long‐term safety of laser and intense pulsed light (IPL) treatments. However, this does not mean that LHR is *“completely risk‐free”*; the likelihood of complications increases, particularly in individuals with darker skin types, when inappropriate parameters are used, or when procedures are performed by untrained individuals. Therefore, in parallel with the growing popularity of LHR in society, it is important for healthcare professionals to have sufficient knowledge regarding the indications, contraindications, potential side effects, and patient counseling aspects of this procedure [22, 23, 24, 25, 26, 27].

The portion of dermatology education in medical schools devoted to the field of cosmetic dermatology is generally insufficient, and the number of class hours allocated to this specialty is limited. There is a mismatch between the widespread need for dermatological care and the time allocated in the curriculum for educating medical students on dermatology topics; this underscores the importance of enhancing dermatology education within the undergraduate medical curriculum. Indeed, it has been reported that dermatology education at the medical school level varies in terms of content and scope, core learning objectives differ by country and institution, and a significant proportion of students find dermatology education inadequate [28, 29, 30, 31]. On the other hand, although LHR is performed in a clinical setting, it is generally perceived in society not as a medical treatment but as a “personal care, aesthetic, and lifestyle” routine. This situation contributes to the preference for sources such as the internet, social media, and friends over scientific literature and professional educational resources during the research process. In our study, 54.4% of medical students reported using the internet to obtain information on the subject, 53.5% used social media, and 53.1% sought information from relatives and friends, while only 25.5% preferred healthcare professionals as their information source. This finding suggests that medical students, who are the future physicians, do not have sufficient access to structured and reliable information regarding a commonly sought cosmetic procedure such as LHR. Ensuring that cosmetic dermatology procedures, particularly laser hair removal, are given sufficient attention during medical school education will foster greater knowledge and awareness in this field and result in the training of physicians who possess an adequate understanding of indications and side effects.

The current situation, namely the inadequacy of education in cosmetic dermatology, is also reflected in the responses provided by the medical students who participated in the study. In the LHR‐K Scale, three statements pertain to the definition of the procedure, two to its application, three to indications, and four to side effects. While one‐third of medical students answered the statements regarding application incorrectly, two‐thirds of the participants answered the statements regarding side effects incorrectly or responded “I don't know”. Furthermore, considering that LHR is primarily performed on women, the high rate of incorrect answers regarding pregnancy and breastfeeding issues closely related to women's health is concerning. Yet, these are the most critical points that should be communicated to the public regarding LHR. The reason these findings go far beyond being merely a *“student survey”* and sound alarm bells for the healthcare system as a whole is the study's subject: *“medical students*.” Medical students are not merely academic students; they are future physicians who will soon enter the community, make clinical decisions, write prescriptions, and be regarded by patients as the “undisputed authority of science and medicine”.

It is understandable for people to feel concerned about situations where they lack sufficient information. In our study, 39.6% of students reported feeling concerned about LHR, and it was found that 30.1% had a high level of concern. A recent cross‐sectional survey conducted on 400 adults at dermatology clinics in Saudi Arabia examined the relationship between the level of knowledge regarding LHR and anxiety related to the procedure. The study demonstrated that a sufficient level of knowledge was associated with lower anxiety levels [32].

In our study, logistic regression analysis revealed that individuals who correctly defined LHR, those who had previously undergone the procedure, and those who used social media to obtain information about LHR were more likely to achieve a sufficient score on the LHR‐K scale.

Possessing accurate knowledge about the basic definition or elements of a subject is crucial for dismantling biases regarding that subject, encouraging more detailed research, and fostering proper understanding. In our study, logistic regression analysis revealed that the likelihood of achieving an adequate score on the LHR‐K scale was higher among those who correctly understood the definition of LHR. Unfortunately, there is a significant lack of information and a prevalence of myths regarding LHR, which is quite popular today. Not knowing what a laser is, defining it as nuclear radiation, X‐rays, or ultrasound waves, believing it causes damage and cancer in various tissues and organs, and thinking it is harmful to children and pregnant women are among the primary myths [33, 34]. There is no evidence‐based scientific data in the literature indicating that LHR causes systemic or oncological harm to human health, damages internal organs, or negatively affects the reproductive system. On the contrary, long‐term follow‐up studies and reviews emphasize that the procedure has a safe profile when performed by qualified professionals, within the appropriate indications, and in the correct manner [24, 27, 33, 35]. For example, in a large‐scale clinical study involving 3606 patients treated with laser therapy and followed for 11.5 years, no major long‐term systemic adverse effects were observed, and any minor side effects (erythema, perifollicular edema) resolved spontaneously within a few days [27].

In our study, the likelihood of achieving a sufficient score on the LHR‐K scale was found to be higher among those who had previously undergone LHR compared to those who had not. This finding can be explained by the fact that individuals who undergo laser hair removal are exposed to pre‐procedural counseling, risk–benefit information, and informed consent processes; additionally, they learn pre‐ and post‐procedural recommendations through practical experience during the procedure. On the other hand, the fact that side effects are mostly temporary when laser hair removal is performed under appropriate conditions, and that serious and permanent complications are rare, may reinforce the perception among experienced individuals that the procedure is relatively safe. Studies reporting high patient satisfaction also support this interpretation. A study conducted by Al Hatmi et al. in Oman reported similar findings to ours [15]. Similarly, in a study by Ağaoğlu et al. involving 1002 patients, it was found that the knowledge levels of those who had previously undergone laser hair removal were higher than those of the group that had not [34].

In our study, the multivariate logistic regression analysis indicated that those who obtained information about LHR from social media were more likely to have sufficient knowledge, whereas no significant difference in knowledge levels was observed between those who obtained information from the internet or from relatives/friends. The inadequacy of the training dedicated to cosmetic dermatology in general medical education has been highlighted previously, and in our study as well, while the internet, social media, and peer groups accounted for high percentages among sources of information, healthcare professionals accounted for a rate that was nearly half as low. In fact, this finding demonstrates how medical students compensate for the difficulties they face in accessing accurate and reliable information through other methods. Among these compensatory methods, social media emerged as a significant factor in ensuring a higher level of information compared to other options. Given this compensatory mechanism, the current result is not particularly surprising, as social media has come to occupy a very special place within general internet access today and has become a highly effective tool for providing individuals with thousands of results in a short period of time. Moreover, social media algorithms analyze users' personal interests and continuously present them with content related to aesthetics, beauty, and personal care. While obtaining health information via social media and the internet offers the advantage of quick access, misinformation, exaggerated marketing language, and the risk of an infodemic are significant concerns on these platforms. Various systematic reviews have shown that health‐related misinformation on social media exists at a significant level, reaching quite high rates in some areas [36, 37, 38]. Specifically in dermatology, it has been reported that a significant portion of social media content is produced by non‐experts, and popular dermatology content across various platforms may be problematic in terms of educational accuracy and quality [39, 40, 41]. Indeed, a comprehensive study conducted in 2020 revealed that only 38% of Instagram accounts featuring popular dermatology content were managed by healthcare professionals, and board‐certified dermatologists accounted for just 4% of these. Furthermore, 27% of these healthcare professionals lacked valid credentials [42]. Cosmetic dermatology is particularly vulnerable to the rapid spread of misinformation; this situation can affect individuals' decision‐making processes, professional credibility, and ultimately public health [11]. Although our study found that individuals obtaining information from social media were more likely to have an adequate level of knowledge on the LHR‐K scale, this result may be due to the fact that these individuals were medical students and were more likely to select and follow reliable health accounts compared to the general public.

## 5 Conclusions

The findings revealed that medical students' level of knowledge regarding LHR was moderate. Given that medical students, as future physicians, will assume the responsibility of informing and guiding their patients on this subject, the fact that their level of knowledge falls short of expectations stands out as a noteworthy finding.

Our study found that students who have undergone LHR, those who plan to undergo it in the future, and those with low levels of concern regarding LHR had significantly higher levels of knowledge. This suggests that personal experience and a positive attitude toward the subject support the knowledge acquisition process. When evaluated in terms of information sources, social media was identified as the primary source through which students access information about LHR. However, the fact that social media contains not only accurate information but also unregulated and unscientific content raises significant concerns regarding the reliability of this source.

Another important issue is the presence of a significant level of misconceptions and myths regarding LHR, both among medical students and in the general public. These findings underscore the need for more comprehensive studies with larger sample sizes on this topic. In this context, it is of great importance to integrate not only theoretical knowledge but also practical training on LHR into medical education programs. This will ensure that students have access to accurate and up‐to‐date information from evidence‐based sources and pave the way for training informed physicians who can help reduce misconceptions in society after graduation.

## Author Contributions

S.M., H.K.E. designed and planned the study; S.S., B.Y.Y. conducted data acquisition; E.A., H.K.E., S.M., S.S., B.Y.Y. performed data analysis and presentation; E.A., H.K.E., S.M., S.S., B.Y.Y., E.A. wrote the initial version of the manuscript, and all authors reviewed the manuscript; E.A., S.M., H.K.E., S.S., E.A. prepared the final version of the manuscript.

## Funding

The authors have nothing to report.

## Disclosure

The author(s) declare that no artificial intelligence (AI) based tools, including generative AI and large language models, were used at any stage of this study, including the study design, data collection, analysis, interpretation of the findings, or the preparation of the manuscript.

## Ethics Statement

Ethical approval for this study was obtained from the Eskişehir Osmangazi University Non‐Interventional Clinical Research Ethics Committee (Decision No. 46, September 26, 2023).

## Consent

Informed consent was obtained from all individual participants included in the study.

## Conflicts of Interest

The authors declare no conflicts of interest.

## Data Availability

The data that support the findings of this study are available on request from the corresponding author. The data are not publicly available due to privacy or ethical restrictions.
